# Gold Nanoparticles in Parkinson’s Disease Therapy: A Focus on Plant-Based Green Synthesis

**DOI:** 10.7759/cureus.54671

**Published:** 2024-02-22

**Authors:** Tsenka Grancharova, Stanislava Simeonova, Bissera Pilicheva, Plamen Zagorchev

**Affiliations:** 1 Department of Medical Physics and Biophysics, Medical University of Plovdiv, Plovdiv, BGR; 2 Research Institute, Medical University of Plovdiv, Plovdiv, BGR; 3 Department of Pharmaceutical Sciences, Medical University of Plovdiv, Plovdiv, BGR

**Keywords:** flavonoids, neuroinflammation, neuroprotection, neurodegenerative, gold nanoparticles, nanozymes, green synthesis, parkinson

## Abstract

Parkinson's disease (PD) is a progressive neurodegenerative disease that affects approximately 1% of people over the age of 60 and 5% of those over the age of 85. Current drugs for Parkinson's disease mainly affect the symptoms and cannot stop its progression. Nanotechnology provides a solution to address some challenges in therapy, such as overcoming the blood-brain barrier (BBB), adverse pharmacokinetics, and the limited bioavailability of therapeutics. The reformulation of drugs into nanoparticles (NPs) can improve their biodistribution, protect them from degradation, reduce the required dose, and ensure target accumulation. Furthermore, appropriately designed nanoparticles enable the combination of diagnosis and therapy with a single nanoagent.

In recent years, gold nanoparticles (AuNPs) have been studied with increasing interest due to their intrinsic nanozyme activity. They can mimic the action of superoxide dismutase, catalase, and peroxidase. The use of 13-nm gold nanoparticles (CNM-Au8®) in bicarbonate solution is being studied as a potential treatment for Parkinson's disease and other neurological illnesses. CNM-Au8® improves remyelination and motor functions in experimental animals.

Among the many techniques for nanoparticle synthesis, green synthesis is increasingly used due to its simplicity and therapeutic potential. Green synthesis relies on natural and environmentally friendly materials, such as plant extracts, to reduce metal ions and form nanoparticles. Moreover, the presence of bioactive plant compounds on their surface increases the therapeutic potential of these nanoparticles. The present article reviews the possibilities of nanoparticles obtained by green synthesis to combine the therapeutic effects of plant components with gold.

## Introduction and background

Parkinson's disease (PD) is a progressive, age-associated neurodegenerative disorder affecting millions of people worldwide. Its incidence is approximately 1% among individuals aged 60 and rises to 5% among those over 85 years old [1]. PD has a silent presymptomatic phase, which makes early detection challenging. The exact cause of PD remains multifactorial, involving factors like aging, environmental influences such as industrialization and pesticide exposure, toxins, and genetic predisposition [2].

PD is characterized by the loss of dopaminergic neurons in the substantia nigra pars compacta (SN) and the presence of Lewy bodies containing misfolded alpha-synuclein aggregates [3]. The reduction in dopaminergic neurons within the SN results in decreased dopamine (DA) production, leading to motor symptoms such as bradykinesia and rigidity [4]. Additionally, non-motor symptoms affect various organ systems and include depression, anxiety, gastrointestinal disorders, olfactory deficits, sleep behavior disorders, cognitive deficits, etc. PD pathogenesis is associated with various factors, including increased oxidative stress, ferroptosis, mitochondrial dysfunction, gut dysbiosis, and neuroinflammation [5-9]. Approximately 5-10% of PD cases are genetically associated with mutations in genes involved in α-synuclein aggregation, dopaminergic neuron loss, defense against oxidative stress, autophagy, mitochondrial function, etc. [10,11].

Oxidative stress plays a crucial role in PD. Various processes in brain physiology contribute to increased reactive oxygen species (ROS) production, including DA metabolism, mitochondria's electron transport chain, and enzymes like monoamine oxidase (MAO), NADPH oxidase, and nitric oxide (NO) [12]. The current medications for PD primarily address motor symptoms and cannot stop the progression of the disease. These treatments mainly focus on replacing DA with drugs such as levodopa (L-DOPA) and DA agonists [13]. Additionally, other medications rely on inhibition of the enzymes MAO and catechol-O-methyl transferase in order to prevent the breakdown of DA. Other therapies for motor symptoms involve DA receptor agonists, anticholinergics, and antiglutamatergics. Iron chelators [14], antioxidants [15], and various neuroprotective substances [16,17] are also being explored as potential therapies for PD.

Current PD treatments face challenges such as rapid degradation, lower bioavailability, and limited brain penetration. Nanotechnology offers a promising solution through various techniques. Proper design of nanoparticles (NPs) could overcome biological barriers and offer alternative administration routes and theranostics abilities [18]. Nowadays, metal ions with the ability to mimic enzyme activities are increasingly used in various NP applications. Gold nanoparticles (AuNP) functionally mimic endogenous antioxidant enzymes and could mitigate oxidative stress [19]. Green methods for NP synthesis, using plant extracts, present an eco-friendly approach for NP preparation, avoiding toxic chemicals and enhancing therapeutic capabilities through bioactive plant compounds [20]. Natural plant-based therapeutic products could counteract numerous pathogenic processes [21], so AuNPs, obtained by green synthesis, could potentially benefit from the therapeutic abilities of both gold and plant compounds.

The aim of the article is to review plant-mediated green synthesis of AuNPs for potential application in PD therapy.

## Review

AuNPs in PD therapy

Role of Nanomedicine in PD Therapy

Oral L-DOPA, the primary treatment for alleviating motor symptoms, undergoes conversion to DA in the brain by aromatic L-amino acid decarboxylase. Unfortunately, L-DOPA faces rapid degradation and decarboxylation before reaching the brain, resulting in a short half-life of approximately 50 minutes and only about 1% of the administered L-DOPA reaching the brain [22]. Other drugs used for PD treatment exhibit difficulty crossing the blood-brain barrier (BBB), unfavorable pharmacokinetics, and limited bioavailability. Reformulation of drugs in NPs presents a promising solution to address these challenges in PD therapy [23]. Using NPs as drug carriers can improve drug biodistribution, protect therapeutic agents from degradation, reduce the necessary drug dosage, and incorporate ligands for enhanced targeted delivery [24]. Another example of a positive role for NPS in PD therapy is neurotrophic factor (NTF)-based therapies. They show promise in treating neurodegenerative diseases by slowing and potentially reversing neurodegeneration [25]. However, their short in vivo half-life, poor pharmacokinetics, and difficulty in penetrating the blood-brain barrier limit their access to neuronal targets. This leads to the need for direct brain delivery in PD patients through intracranial surgery. NP-based delivery systems have attracted attention for their ability to improve NTF delivery efficiency. NP agents that demonstrate promising improvements in NTF delivery are summarized by Bondarenko and Saarma [26]. NTFs can also serve as targeting ligands, for example, by using nerve growth factor (NGF) receptor-mediated endocytosis. Using this strategy, Hu et al. [27] investigated NGF-conjugated AuNP composites that promote neuronal uptake. Their research showed that these NP agents loaded with plasmid DNA effectively suppressed α-synuclein expression. Additionally, well-designed NPs possess multifunctional theranostic potential, enabling both therapy and the ability to diagnose and monitor the delivery of therapeutic compounds to the brain. Intranasal delivery of drugs directly to the brain through the olfactory nerve also benefits NPs by improving availability, accuracy, and controlled release [28].

**Figure 1:**
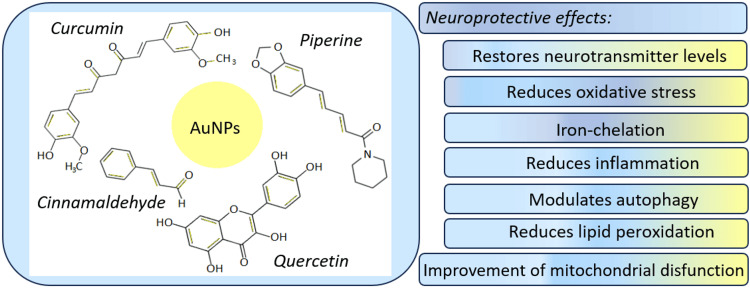
Schematic representation of green-synthesized AuNPs and their neuroprotective properties.

Despite various techniques for NP preparation, green synthesis has led to increased interest due to its simplicity and therapeutic potential. Traditional methods for NP synthesis are often expensive, require specialized equipment, and can have a negative impact on the environment due to the use of toxic and harmful chemicals. In contrast, green synthesis employs natural and eco-friendly materials, such as plant extracts, to reduce metal ions to a lower valence state. This approach eliminates the need for harmful chemicals and toxic solvents [20]. Furthermore, the plant phytochemicals not only act as reducing agents but also enhance the therapeutic capabilities of NPs resulting from the incorporation of bioactive plant compounds on the NPs' surface [29] (Figure 1).

Toxicity of AuNPs

Although bulk gold is non-toxic, AuNPs exhibit distinct properties such as enhanced reactivity, a larger surface area relative to volume, and the capability for surface modifications. These features make them valuable in nanomedicine but can also impact NP excretion rates, potentially leading to toxicity. The toxicity of AuNPs is influenced by factors including their size, shape, surface charge, and synthesis methods, as well as the dosage and administration routes [30]. Yao et al. [31] summarized data from over 20 studies involving more than 700 patients to assess the toxicity of AuNPs used in medical interventions. The findings from these studies indicate that AuNPs have generally shown good safety profiles with minimal adverse events. Kumthekar et al. [32] conducted a phase 0 clinical trial (NCT03020017) to determine the safety, pharmacokinetics, and biodistribution of AuNPs composite in patients with recurrent glioblastoma. Long-term Au retention was observed for two patients' tumors, maintaining 41% and 81% of initial levels after 159 and 174 days, respectively. In another study, Goel et. al. [33] investigated the short-term and long-term biodistribution and elimination of AuNPs conjugated with targeting TNF-α. The results showed that utilizing a combination of tumor-targeting ligands on AuNP is a strategy to improve the safety of nanomedicines. Longer-term animal studies showed that the liver gradually cleared the accumulated Au over time, with approximately 35% of the injected AuNPs (~43 µg) detectable in the liver and spleen at day 120.

Balfourier et al. [34] monitored the biotransformations of AuNPs in primary human fibroblasts over two to six months. They discovered that cells can degrade 4- to 22-nm GNPs, mediated by NADPH oxidase, with smaller NPs degrading faster. Additionally, gold re-crystallizes into biomineralized nanostructures, forming 2.5-nm particles arranged into nanoleaves that resemble in vivo autosomes. Despite these findings, the limited number of trials, small sample sizes, and uncertainties about long-term gold accumulation pose challenges in applying AuNPs clinically, necessitating further research into their long-term toxicity.

AuNPs in PD Therapy

In recent years, AuNPs have found applications as catalysts in chemical reactions, mimicking the activities of natural enzymes. They exhibit superoxide dismutase-like activities by eliminating O2, catalase-like activities by reducing H2O2 to water and oxygen, and peroxidase activity by catalyzing oxidation reactions with peroxides. Gold-based nanoformulation, 13-nm AuNPs CNM-Au8® in drinkable bicarbonate solution, is being investigated for the treatment of neurodegenerative disorders, including PD (NCT03815916) [31]. CNM-Au8® demonstrates catalytic properties that help alleviate cellular energy deficits and reduce oxidative stress. The AuNPs have shown the ability to convert NADH into NAD+ [35], a crucial factor in cellular energy production. In animal models of demyelination, CNM-Au8® has demonstrated promise in enhancing remyelination and improving motor functions [36]. Phase 2 clinical trials, REPAIR-MS, NCT03993171, and REPAIR-PD, NCT03815916, investigated the effect of CNM-Au8 on brain energy metabolites and evaluated its safety [37]. The results align with preclinical findings, indicating a positive modulation of brain energy metabolism in multiple sclerosis or PD patients. The two disease cohorts showed a 10.4% increase in the brain NAD+/NADH ratio compared to baseline following 12 weeks of daily oral CNM-Au8 treatment. No severe treatment adverse events were reported, and CNM-Au8 was well-tolerated in all participants. Furthermore, the unique optical properties of AuNPs are being explored for their potential use in sensitive detection systems [38].

Green synthesis of AuNPs for PD therapy

Curcuma longa-Mediated AuNPs

*Curcuma longa* is widely investigated for its antioxidant and anti-inflammatory properties. The diverse pharmacological actions of *C. longa* are primarily associated with its polyphenolic compound curcuminoids, particularly curcumin. In various models of PD, curcumin has shown neuroprotective effects, preventing DA loss by exhibiting iron-chelating activity, reversing oxidative stress, and improving mitochondrial dysfunction. In 1-methyl-4-phenyl-1,2,3,6-tetrahydropyridine (MPTP)-induced toxicity, curcuminoids showed their neuroprotective potential by preventing neurodegeneration, reducing pro-inflammatory responses and microglial activation, and managing oxidative stress factors [39]. Another study [40] demonstrated that *C. longa* administered orally in a 6-hydroxydopamine (6-OHDA)-induced PD model significantly mitigated oxidative stress and reduced inflammatory symptoms in rats. This treatment led to an increase in SNAP-25 and BDNF levels while lowering α-synuclein levels, improving motor skills and memory, and minimizing anxiety- and depression-like behaviors. Additionally, curcumin's protective effects against 6-OHDA, due to its iron-chelating capabilities, prevent the iron-induced degeneration of nigral dopaminergic neurons [41].

Nellore et al. [42] studied the synthesis of AuNPs using *C. longa* root extract and their effect on 1-methyl-4-phenylpyridinium (MPP+)-induced cytotoxicity in PC-12 cells. Their findings revealed that *C. longa*-AuNPs possessed cytoprotective properties against toxin-induced oxidative stress. Pre-treatment in PC-12 cells with *C. longa*-AuNPs demonstrated inhibitory effects on lipid peroxidation, preventing the toxin-induced membrane disruption, and increasing cell viability in a dose-dependent manner. Additionally, *C. longa*-AuNPs significantly counteracted the reduced antioxidant enzyme activity and mitochondrial complex I activity caused by MPP+, thereby reversing the induced toxicity.

Hibiscus syriacus-Mediated AuNps

*Hibiscus syriacus* Linn., a member of the Malvaceae family, exhibits diverse biological properties, including anticancer, antioxidant, antidepressant, antiaging, and neuroprotective activities. Scopoletin from *H. syriacus* demonstrated MAO inhibitory activity [43]. Zhang et al. [44] investigated the neuroprotective effects of polyphenols and fatty acids from *H. syriacus* on lipopolysaccharides (LPS)-induced inflammation in SH-SY5Y cells. The findings revealed that polyphenols from plants exhibited a neuroprotective effect in SH-SY5Y cells. PCR analysis indicated improvement in the mRNA expression of synapse-related genes and neurotrophic factors. Additionally, the polyphenols reduced ROS levels, neuroinflammation, and oxidative stress induced by H_2_O_2_ and increased antioxidant enzyme activities. Kim et al. [45] investigated the antidepressant and neuroprotective effects of a root bark extract of *H. syriacus* and their molecular mechanisms in a mouse model of depression. Their findings revealed that the administration of *H. syriacus* significantly alleviated depression-like behavior, mitochondrial oxidative stress, and neuronal inflammation.

Xu et al. [46] investigated AuNPs synthesized by an extract of *H. syriacus* and evaluated their anti-inflammatory effects in an LPS-induced inflammation model in RAW264.7 cells. The results demonstrated that *H. syriacus*-AuNPs reduced pro-inflammatory cytokines and restored mitochondrial function by reversing the changes in mitochondrial membrane potential, decreasing ROS, and recovering ATP levels. The study indicated that *H. syriacus*-AuNPs could potentially prevent diseases associated with progressive inflammation and mitochondrial dysfunction by inducing autophagy. In addition, *H. syriacus*-AuNPs showed better treatment ability compared to *H. syriacus* at the tested concentrations.

Hypericum hookerianum-Mediated AuNPs

The species of Hypericum, especially *H. perforatum* (commonly known as St. John’s wort), is widely used as an antidepressant. Hond et al. [47] explored the neuroprotective potential of twenty known compounds isolated from *H. hookerianum*, including flavonoids, lignans, xanthones, chalcone, chromone C-glycoside, cinnamic acid derivatives, phloroglucinols, and stilbene. Four of these compounds, quercetin, 4-hydroxy-2,6,4'-trimethoxydihydrochalcone, ethyl-4-methoxy-trans-cinnamate, and 5,7-dihydroxy-2-(1-methylpropyl)chromone-8-β-D-glucopyranoside, displayed significant neuroprotection against glutamate-induced toxicity in HT-22 cells, with 4-hydroxy-2,6,4'-trimethoxydihydrochalcone showing the highest efficacy. Furthermore, piperitol, sesamin, and chipericumin D demonstrated neuroprotective effects in the 6-OHDA toxicity model in SH-SY5Y cells, with sesamin exhibiting the most prominent activity.

Subakanman et al. [48] compared the effectiveness of ethanolic extracts of *H. hookerianum* and *H. hookerianum*-synthesized AuNps in treating haloperidol-induced PD in mice. Various tests, such as behavioral tests (rota rod, gait analysis, and wire hang), along with neurotransmitter analysis (DA and glutamate), were used to evaluate their neuroprotective properties against PD symptoms. The study showed that both *H. hookerianum* extract and *H. hookerianum*-AuNps improved motor function, reduced lipid peroxidation, and restored neurotransmitter levels, with *H. hookerianum*-AuNps showing better anti-PD effects.

Moutan Cortex-Mediated AuNps

Kim et al. [49] demonstrated the protective effects of Moutan Cortex Radicis (MCR), the root bark of *Paeonia suffruticosa* (Moutan peony, *Paeonia moutan*), against MPTP-induced PD-like symptoms. In a rat primary mesencephalic culture, MCR protected dopaminergic neurons, inhibited mitochondrial dysfunction, increased DA availability, and improved motor symptoms. MCR also elevated the Bcl-2/Bax ratio, suppressed cytochrome C release, and inhibited caspase-3 activation, indicating potential benefits for treating neurodegenerative PD and PD-like diseases.

Xue et al. [50] investigated the synthesis of AuNPs using *P. moutan* root extract (PM-AuNPs) and their efficacy against PD in a mouse model. PM-AuNPs treatment significantly scavenged reactive oxygen and inhibited NO and inflammatory cytokine synthesis. Results showed that PM-AuNPs improved motor coordination in PD-induced mice. PM-AuNPs treatment reduced COX-2 expression, preventing COX-2-induced dopaminergic neurodegeneration. These NPs also exhibited efficacy against induced neuroinflammation in BV2 murine microglial cells. In vitro results showed that PM-AuNPs efficiently scavenged reactive oxygen species and decreased inflammatory cytokine levels in microglial cells, establishing PM-AuNPs as a potential treatment for PD.

Cinnamomum-Mediated AuNPs

The study of Ramazani et al. [51] explored the therapeutic potential of Cinnamomum species, particularly *Cinnamomum verum* and *Cinnamomum cassia*, and cinnamaldehyde on 6-OHDA-induced PC12 cytotoxicity as an in vitro model of PD. Cinnamaldehyde, a prominent antioxidant component, underpins many of the biological activities of cinnamon. The treatments substantially enhanced cell viability and reduced ROS levels in 6-OHDA-exposed cells. They also effectively suppressed the activation of the p44/42 pathway, a mitogen-activated protein kinase associated with ROS-induced apoptosis. Furthermore, the treatments increased survivin expression, an inhibitor of apoptosis, while decreasing cytochrome C levels.

Ling et al. [52] explored the potential therapeutic effects of AuNPs synthesized from *C. verum* in an MPTP-induced PD rat model. The administration of *C. verum*-AuNPs was found to reduce oxidative stress and motor abnormalities induced by MPTP in PD rats. Various behavioral tests were used to assess motor function impairments in the rat models. *C. verum*-AuNPs treatment effectively restored the behavioral deficits caused by MPTP in the PD rats, suggesting their potential to mitigate motor impairments associated with PD.

Piperine-Mediated AuNPs

Piperine, a compound found in black pepper, has shown potential in both preclinical and clinical trials for treating various diseases. Clinical research has explored its protective and therapeutic effects on conditions like microbial infections, hypertension, neurological disorders, cancer, diabetes, cardiovascular problems, and reproductive issues [53]. The broad range of actions by piperine is attributed to its ability to interact with multiple molecular targets, including kinases, inflammatory cytokines, receptors, central nervous system targets, MAO-B, est. Piperines contribute to neuroprotection by reducing inflammation, oxidative stress, and mitochondrial damage. Oral piperine treatment improved cognitive learning in PD mouse models and demonstrated antioxidant properties [54].

Sharma et al. [55] used rotenone and iron toxicity as PD animal models to validate the neuroprotective effect of quercetin in combination with piperine. Rotenone disrupts mitochondrial complex 1, leading to increased oxidative stress, neuroinflammation, neurotransmitter and motor function changes, thus mimicking PD disease. The application of quercetin, a natural polyphenolic flavonoid, and piperine treatments, individually and combined, significantly improved rat locomotor activity and grip strength behavior, and attenuated behavioral alteration. Additionally, piperine and quercetin reduced lipid peroxidation, restored GSH levels and mitochondrial complex I and IV activities, attenuated neuroinflammatory markers, and restored DA, norepinephrine, and serotonin levels.

Srivastav et al. [56] synthesized piperine-coated AuNPs and assessed their protective effect on paraquat (PQ) toxin-induced PD models in Drosophila melanogaster and SH-SY5Y cells. Piperine-AuNPs demonstrated effectiveness in suppressing oxidative stress and mitochondrial dysfunction, preventing apoptotic cell death in PQ-treated flies. Similarly, they protected SH-SY5Y cells from PQ-induced toxicity by preserving mitochondrial membrane potential. Piperine-AuNPs supplementation mitigated PQ-induced apoptosis through the regulation of Jnk and caspase-3 activity, and also improved locomotor function and lifespan. In comparison to piperine and control AuNPs, piperine-AuNPs displayed better cytoprotective effects against PQ-induced toxicity in SH-SY5Y Cells.

Bacopa monnieri-Mediated AuNPs

*Bacopa monnieri *(BM), belonging to the family Scrophulariacea, is a well-known plant with therapeutic potential in neurological disorders and memory-related diseases [57]. Current evidence on BM indicates that the mechanism of antioxidant neuroprotection relies on redox and enzyme induction, metal ion reduction, free radical scavenging, lipid peroxidation inhibitory activities, acetylcholinesterase inhibition and/or choline acetyltransferase activation, β-amyloid reduction, increased cerebral blood flow, and neurotransmitter modulation [58]. The triterpenoid saponins and their bacosides are responsible for Bacopa’s ability to enhance nerve impulse transmission. The bacosides, a significant component of BM, aid in the repair of damaged neurons by enhancing kinase activity, neuronal synthesis, restoration of synaptic activity, and ultimately, nerve impulse transmission.

Investigation of the neuroprotective and neurorescuing properties of BM extract finds a significant recovery in behavioral parameters, DA level, glutathione level, lipid peroxides, and nitrite level, against the MPTP-induced mice model of PD [59]. Treatment with BM before or after MPTP administration has a protective effect on dopaminergic neurons by either increasing DA synthesis or inhibiting DA degradation. In a similar study [60], pre-treatment with BM significantly decreased the level of alpha-synuclein in PD, induced in rats by rotenone. No significant difference was observed between the pre-treatment with BMand the L-dopa treatment group in the hippocampus, substantia nigra, and striatum regions. BM led to a significant decrease in the levels of pro-inflammatory cytokines and oxidative stress in the hippocampus, substantia nigra, striatum, cortex, and brain stem regions.

Bommavaram et al. [61] explored the antioxidant potential of AuNPs synthesized using BM plant extract in aluminum-induced oxidative stress in mice. The administration of BM-AuNPs resulted in a significant reduction in lipid peroxidation and an increase in antioxidant enzyme activity.

Mucuna pruriens-Mediated AuNPs

*Mucuna pruriens *(MP), a member of the Fabaceae family, is known for its diverse therapeutic properties, including antioxidative, anti-inflammatory, anti-epileptic, anti-microbial, anti-cancer, anti-anemic, and antihypertensive effects [62]. Key constituents like L-DOPA, ursolic acid, and betulinic acid contribute to MP anti-PD activity.

Arulkumar and Sabesan [63] explored the effect of MP extract and MP-AuNPs on MPTP-induced neurotoxicity. The MPTP application induced impaired rotarod performance, altered locomotor activity, increased crossing time on the beam, and a significantly reduced hang time. Treatment with MP and MP-AuNPs significantly improved these deficits, which were correlated with elevated DA levels.

Future prospects

Many plants provide beneficial health effect for treating PD's symptoms. By combination with gold, these plants can potentially enhance their therapeutic effect. Some substances of plant origin show dopaminergic activity [64], ability to target α-synuclein aggregation [65], inhibit monoamine oxidases [66,67] or have antioxidant properties [68,69]. Many fruits, especially berries, also show potential to improve symptoms in patients with the PD [70-72]. Green synthesis could be successfully used to produce nanoparticles by fruit extracts [73]. The investigation of similar nanoparticles in the prevention and therapy of PD will also be of interest.

## Conclusions

PD presents a significant health challenge with its complex etiology involving genetic, environmental, and age-related factors. Current treatments, primarily addressing motor symptoms, face challenges such as rapid degradation, limited bioavailability, and poor brain penetration. The application of well-designed NPs holds the potential to improve drug biodistribution, protect therapeutic agents, and enhance targeted delivery, thereby opening new horizons for more effective and precise PD treatment strategies. The use of green synthesis of AuNPs not only simplifies the preparation process but also incorporates bioactive plant compounds, enhancing therapeutic capabilities. The innovative approach of AuNPs combined with various plant compounds presents an avenue for future research in PD prevention and therapy.
